# Urinary Biomarkers of Aminoglycoside-Induced Nephrotoxicity in Cystic Fibrosis: Kidney Injury Molecule-1 and Neutrophil Gelatinase-Associated Lipocalin

**DOI:** 10.1038/s41598-018-23466-4

**Published:** 2018-03-23

**Authors:** Stephen J. McWilliam, Daniel J. Antoine, Andrea L. Jorgensen, Rosalind L. Smyth, Munir Pirmohamed

**Affiliations:** 1 Department of Women’s and Children’s Health, University of Liverpool, Merseyside, United Kingdom; 2 MRC Centre for Inflammation Research, University of Edinburgh, Edinburgh, United Kingdom; 3 Department of Biostatistics, University of Liverpool, Liverpool, Merseyside, United Kingdom; 4 University College London, Great Ormond Street Institute of Child Health, London, United Kingdom; 5 Department of Molecular and Clinical Pharmacology, and MRC Centre for Drug Safety Science, University of Liverpool, Liverpool, Merseyside, United Kingdom

## Abstract

Aminoglycosides are commonly used for the treatment of pulmonary exacerbations in patients with cystic fibrosis (CF). However, they are potentially nephrotoxic. This prospective observational cohort study aimed to investigate the potential validity of two urinary renal biomarkers, Kidney Injury Molecule-1 (KIM-1) and Neutrophil Gelatinase-associated Lipocalin (NGAL), in identifying aminoglycoside-induced nephrotoxicity in children with CF. Children and young adults up to 20 years of age with a confirmed diagnosis of CF were recruited from ten United Kingdom hospitals. Participants provided urine samples for measurement of KIM-1 and NGAL concentrations, at baseline, at regular outpatient appointments, and before, during and after exposure to clinically-indicated treatment with the aminoglycoside tobramycin. 37/158 patients recruited (23.4%) received at least one course of IV tobramycin during the study. The median peak fold-change during tobramycin exposure for KIM-1 was 2.28 (IQR 2.69) and 4.02 (IQR 7.29) for NGAL, in the absence of serum creatinine changes. Baseline KIM-1 was positively associated with cumulative courses of IV aminoglycosides (R^2^ = 0.11; β = 0.03; p < 0.0001). KIM-1, in particular, may be a useful, non-invasive, biomarker of acute and chronic proximal tubular injury associated with exposure to aminoglycosides in patients with CF, but its clinical utility needs to be further evaluated in prospective studies.

## Introduction

Cystic fibrosis (CF) is characterised by secondary bacterial lung infections and pulmonary colonisation, often by resistant organisms, in particular *Pseudomonas aeruginosa*. Aminoglycosides have good efficacy against *P*. *aeruginosa* and are commonly used to treat pulmonary exacerbations in CF, usually in combination with a beta-lactam antibiotic^1^. However, aminoglycosides are potentially nephrotoxic, causing targeted toxicity to proximal tubule epithelial cells. Despite risk reduction strategies, current or recent aminoglycoside exposure is strongly associated with acute kidney injury (AKI) in children with CF^2–4^. One recent study identified AKI in 20% of courses of aminoglycoside exposure in children with CF^5^. Chronic renal impairment related to cumulative aminoglycoside exposure has been reported in 31–42% of an adult CF patient cohort^6^.

Renal function is currently evaluated clinically using serum creatinine measurement. However, creatinine elevation is only seen when significant kidney damage has occurred^7^, which means that identification of AKI is frequently late, and the degree of damage may be underestimated^8^. To identify patients at increased risk of renal impairment from aminoglycosides, there is a need for the development of improved biomarkers that not only reflect the site of toxicity, but can identify damage at an earlier stage than currently possible. This would, in turn, allow for treatment adjustment and the avoidance of any further decline in renal function.

This study investigates the potential of two urinary biomarkers, Kidney Injury Molecule-1 (KIM-1) and Neutrophil Gelatinase-associated Lipocalin (NGAL), in the identification of aminoglycoside-induced nephrotoxicity in children and young adults with CF. KIM-1 is the only proximal tubule specific biomarker to have been formally qualified by regulatory agencies for use in preclinical drug development^9^, and outperforms other markers in animal models of aminoglycoside-induced nephrotoxicity^10^. NGAL has also shown promise in animal models of nephrotoxicity^11^. We have previously demonstrated the validity of both in identifying acute kidney injury induced by aminoglycosides in pre-term neonates^12^. Our aims were three-fold: (1) to determine whether urinary KIM-1 and NGAL would be elevated during exposure to tobramycin in children with CF; (2) to determine whether the estimated glomerular filtration rate (eGFR) decreases with cumulative aminoglycoside exposure; and (3) whether any association exists between baseline urinary biomarker concentration and previous exposure.

## Results

### Demographics

A total of 158 children and young adults with CF were recruited to the study. Summary characteristics are presented in Table 1. Thirty-seven of the 158 patients (23%) received at least one course of treatment with IV tobramycin during the 12–24 months follow-up in the study. Fifteen patients were lost to follow-up. Of these, seven withdrew consent during the follow-up period, seven transferred to adult services, and one died. For those withdrawing consent, data was included up to the point of withdrawal, whilst data up to the date of loss to follow-up was included for all others lost to follow-up.

**Table 1:** Baseline characteristics of children with CF recruited to the URBAN CF study.

Demographic		URBAN CF cohort (n = 158)
Age (years) [mean (standard deviation)]		7.35 (5.1)
Sex [number (%)]	Male	78 (50)
	Female	80 (50)
Ethnicity [number (%)]	White	156 (99)
	Other	2 (1)
Genotype [number (%)]	Homozygous DF508	84 (53)
	Heterozygous DF508	63 (40)
	Other	10 (6)
Height (cm) [mean (standard deviation)]		117.6 (33.9)
Weight (kg) [mean (standard deviation)]		26.5 (16.5)
Previous exposure to IV aminoglycoside [number (%)]		86 (54)
Previous exposure to colistin [number (%)]		91 (58)
Previous ototoxicity [number (%)]		2 (1)
Existing renal disease [number (%)]		2 (1)
Existing CF related diabetes [number (%)]		4 (3)

### Associations with baseline biomarkers

**Figure 1:**
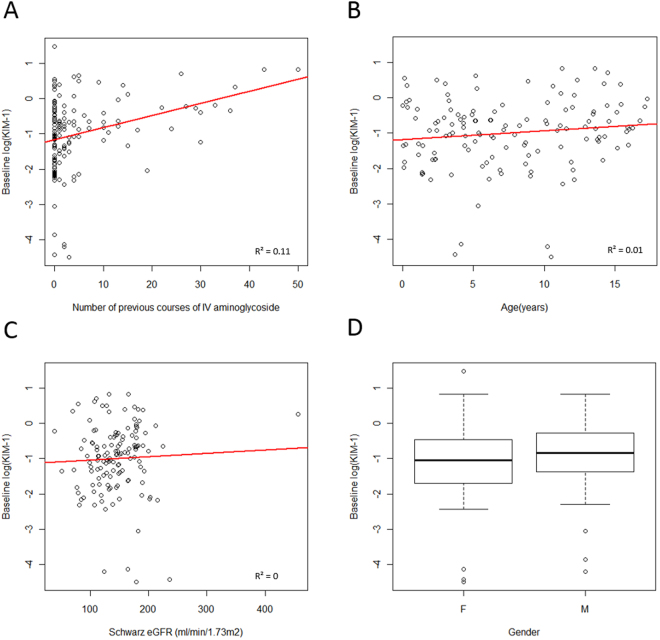
Baseline urinary KIM-1 values. Figures show log(KIM-1) against the number of previous courses of intravenous aminoglycosides (**A**), age (**B**), Schwartz eGFR (**C**) and gender (**D**). Box and whisker plots, present first and third quartile and the median (circles represent outliers).

**Figure 2:**
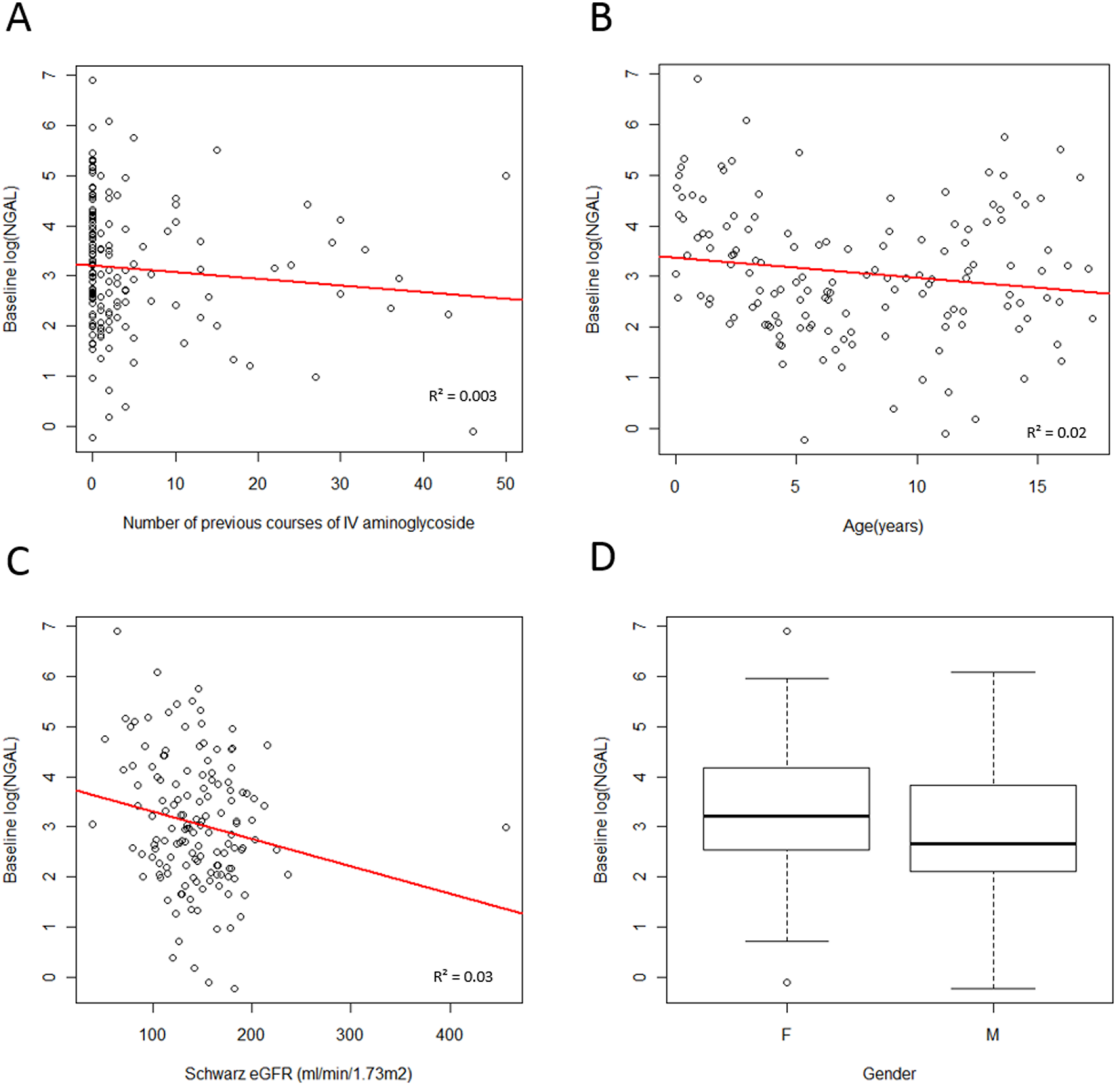
Baseline urinary NGAL values. Figures show log(NGAL) against the number of previous courses of intravenous aminoglycosides (**A**), age (**B**), Schwartz eGFR (**C**) and gender (**D**). Box and whisker plots, present first and third quartile and the median (circles represent outliers).

In the multiple regression model, log-baseline KIM-1 was associated only with the number of previous IV aminoglycoside courses (p < 0.0001; R^2^ = 0.11; β = 0.03 (0.02, 0.05)). A scatterplot of log-baseline KIM-1 against the number of previous courses (Fig. 1A) demonstrated that log-baseline KIM-1 levels increased with increased previous exposure. As expected, number of courses of aminoglycoside was correlated with age (Pearson’s correlation coefficient = 0.51). However, the association between number of aminoglycoside courses and KIM-1 remained even when adjusting for the effect of age in the multiple regression model. For log-baseline NGAL, only gender was found to be statistically significant with males having lower values (p = 0.02; R^2^ = 0.03; β = −0.48 (−0.89, −0.07)) (Fig. 2D).

**Figure 3:**
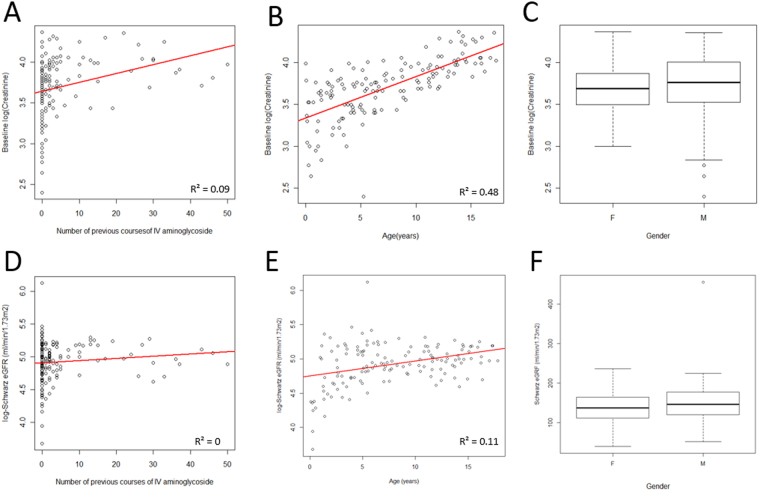
Baseline serum creatinine and eGFR values. Figures show log(serum creatinine) (**A**–**C**) and log(eGFR) (**D**–**F**) against the number of previous courses of intravenous aminoglycosides (**A** and **D**), age (**B** and **E**) and gender (**C** and **F**). Box and whisker plots, present first and third quartile and the median (circles represent outliers).

In the case of serum creatinine, age (p < 0.00001; R^2^ = 0.48; β = 0.05(0.04, 0.06)) remained in the multiple regression model and so was found to be significantly associated with log-baseline creatinine. A scatterplot (Fig. 3B) demonstrated that log-baseline creatinine increased with age. In terms of log-baseline eGFR, only age (p = 0.0004; R^2^ = 0.11; β = 0.02 (0.01, 0.03)) was retained in the final model (Fig. 3E).

### The relationship between elevations in urinary biomarkers and aminoglycoside exposure

**Figure 4:**
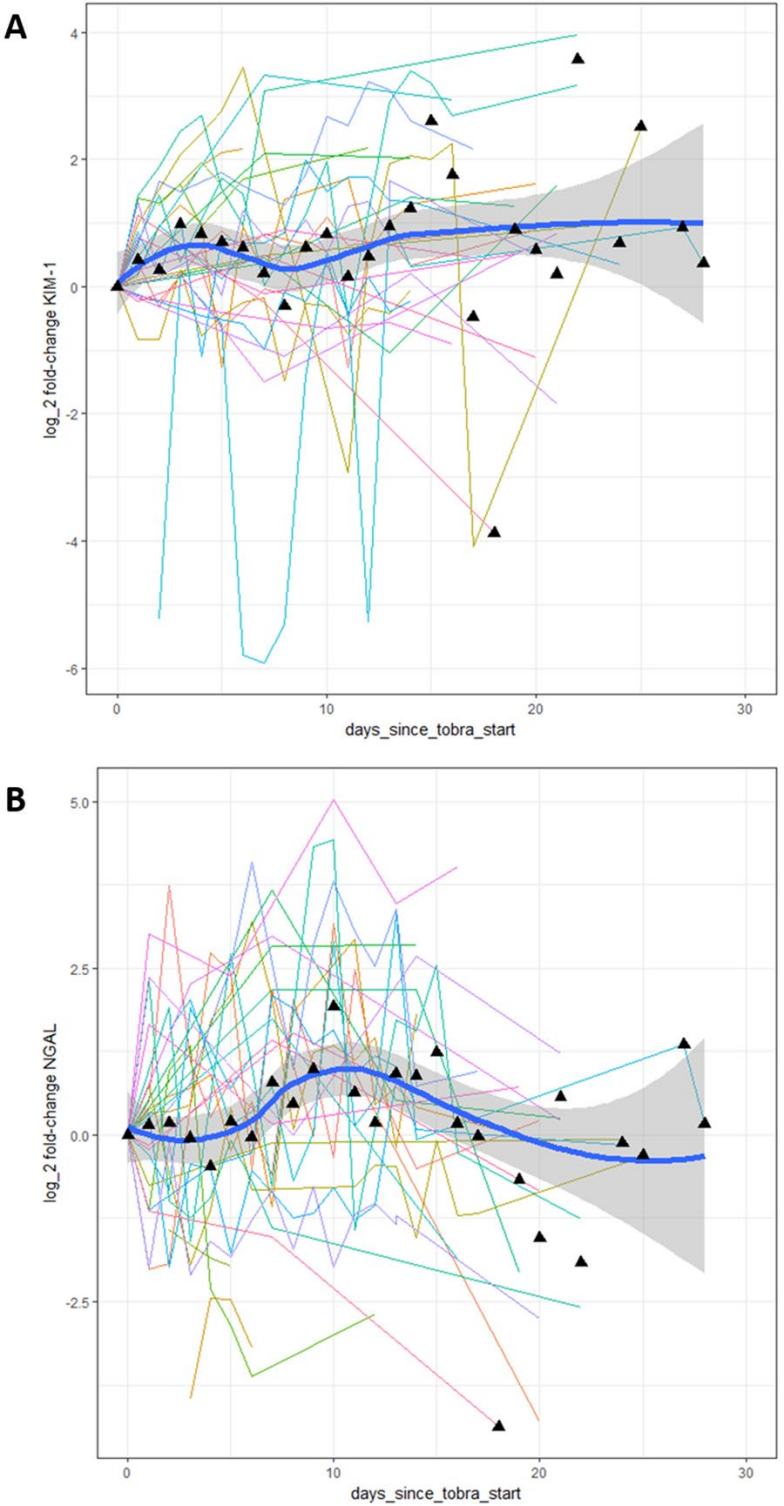
Fold change in biomarker values during and after exposure to tobramycin. Figures show log (base 2) of fold change for KIM-1 (**A**) and NGAL (**B**). Each line represents a different individual receiving tobramycin (usually lasting 14 days). Daily mean value (black triangles), and a mean line with 95% CI are plotted to demonstrate the overall trend of the data.

For both KIM-1 and NGAL, the distribution of peak fold-change was skewed. Median peak fold-change during tobramycin exposure for KIM-1 was 2.28 (IQR 2.69), and 4.02 (IQR 7.29) for NGAL (n = 37). Median peak fold-change during tobramycin exposure for serum creatinine was 1.07 (IQR 0.18) (n = 24, serum creatinine concentrations during tobramycin exposure not available for 13 participants). No patient developed AKI (as defined by the KDIGO criteria^13^). Longitudinal profile plots (Fig. 4) demonstrated a high degree of intra- and inter-individual variability during exposure to tobramycin. However, the mean trend illustrated by the lowess plot suggested that both biomarkers increased in urine during exposure to tobramycin. KIM-1 appeared to increase earlier, with a peak at 3–5 days, with NGAL peaking later, at 9–11 days. After completing tobramycin (usually at 14 days), the mean trend suggests that NGAL returned to its pre-tobramycin (day 0 sample) value, whereas KIM-1 remained elevated.

## Discussion

In this paper, we present data collected from a prospective study demonstrating that, in children and young adults with CF, acute changes are observed in both urinary KIM-1 and NGAL during exposure to IV tobramycin. Furthermore, we show that baseline KIM-1 concentration increases with cumulative aminoglycoside exposure. Baseline urinary NGAL was associated with gender, and baseline serum creatinine was associated with age, but neither was associated with cumulative aminoglycoside exposure. Estimated GFR was not found to be correlated with previous aminoglycoside exposure.

In accordance with our previous study in preterm neonates^12^, elevations in both KIM-1 and NGAL were observed during exposure to tobramycin. Exploratory plots suggest that KIM-1 rises earlier and reaches a peak at 3–5 days, whereas NGAL rises later and reaches a peak at 9–11 days. This finding is consistent with preclinical studies^10^ and our previous report in neonatal infants demonstrating the sensitivity of KIM-1^12^. Furthermore, mean trend plots suggest that KIM-1 remained elevated throughout tobramycin treatment and for some time afterwards, whereas NGAL appeared to return to its pre-tobramycin level at the end of treatment. Given the large degree of inter- and intra-individual variability it is clear that not all participants follow the same trend, and the variability is such that it would not be appropriate to come to concrete conclusions from these observations. Indeed, no patient developed AKI (as defined by the KDIGO criteria^13^) during our study, which is not surprising since we were not powered to detect it. Therefore it is not possible to comment on the predictive value of either biomarker for AKI. However, our findings are consistent with a recent randomised trial of morning versus evening administration of IV tobramycin in children with CF^14^, and a published abstract that also reported elevated KIM-1 concentrations in children with CF receiving aminoglycosides^15^. Furthermore, our findings are consistent with pre-clinical studies which have highlighted the sensitivity of KIM-1 in detecting renal injury^10^.

A novel finding from this study is that baseline KIM-1 in children with CF was associated with cumulative lifetime exposure to IV aminoglycosides. We also demonstrated a slight increase in median KIM-1 with age, in contrast to our previously published reference ranges in healthy children^16^. Indeed, several children with CF aged 13–16 years had baseline KIM-1 values above the upper limit of normal for their age (1.10 ng/mgCr). Our data suggest this elevation may be secondary to cumulative aminoglycoside exposure in these patients. A previous study measuring KIM-1 in children with CF also found a significant correlation with cumulative exposure to aminoglycosides^17^. Our data suggest that KIM-1 becomes elevated during acute episodes of proximal tubule epithelial cell death caused by aminoglycoside exposure, but then remains elevated. Its chronic elevation suggests that the proximal tubule does not fully recover from the acute event, and that KIM-1 is playing a role in this longer term response to toxicity.

This interpretation is supported by a growing body of literature which suggests that KIM-1 may be a useful marker for the development of chronic kidney disease (CKD)^18^. Urinary KIM-1 has been shown to track disease progression and regression in both IgA nephropathy^19–24^ and diabetic nephropathy^25,26^. In AKI, KIM-1 plays an important protective role, promoting tubular epithelial cell regeneration^27^, and conferring a phagocytic phenotype on epithelial cells which may be important in clearing kidney tubules of apoptotic and necrotic cell debris^28^. In CKD it has been hypothesised that KIM-1 may lead to excessive epithelial cell proliferation and have a role in the development of tubular fibrosis^27^.

Baseline urinary NGAL concentration was not associated with previous aminoglycoside exposure. It was associated with sex, which is consistent with our findings in a cohort of healthy children^16^, and with published literature in other populations^29–33^. Compared to healthy children^16^, baseline NGAL is elevated in children with CF, with a number having concentrations above the upper limit of normal. In theory NGAL has promise as a biomarker of aminoglycoside-induced nephrotoxicity as it is taken up by proximal tubule epithelial cells via the same megalin-mediated endocytosis as aminoglycosides^34^. However, NGAL is not renal specific and has consistently been demonstrated to be elevated in inflammatory conditions and sepsis^35–37^. This may explain why baseline NGAL is elevated in patients with CF compared to healthy controls. A previous study suggested it does not become further elevated during pulmonary exacerbations^37^. However, the possibility that any elevation of urinary NGAL seen in patients with CF may be due to sepsis cannot be excluded.

Baseline serum creatinine was strongly correlated with age as has been well described previously in children^38^. Estimated GFR was calculated using the Schwartz formula. No correlation was found between eGFR and cumulative lifetime exposure to aminoglycosides. This contrasts with observations made in a cohort of adult patients with CF^6^ where both measured creatinine clearance, and eGFR calculated using the Cockroft-Gault formula^39^, were demonstrated to decrease with increasing lifetime exposure to aminoglycosides. However, the present findings are in agreement with a previous study in a French paediatric CF cohort^40^. In our cohort, it may be that too few patients were exposed to sufficient courses of aminoglycosides for an effect on eGFR to be seen. It is also conceivable that the observed elevation in urinary KIM-1 in our younger cohort reflects chronic damage of the proximal tubule epithelial cells which, with time, will result in a global impairment of renal function and a reduction in eGFR. Advances in management, especially the advent of once daily dosing of aminoglycosides, may also account for differences between the current paediatric cohort, and an adult cohort recruited around a decade before^6^. It is also important to note that whilst the Schwartz formula^41^ is widely used for the calculation of eGFR in children, it is not validated for use in children with CF^42^, although it has been used in this population previously^40,43^. Indeed, estimates of GFR which depend on serum creatinine may overestimate renal function in CF due to the reduced muscle mass in these patients^42^. Therefore any further investigation should involve measurement of GFR by a ‘gold standard’ test (such as Iohexol clearance) alongside calculation of eGFR.

Interpreting our findings in the light of existing literature, we hypothesise that elevations in KIM-1 and NGAL during aminoglycoside exposure, in the absence of elevations in serum creatinine, represent renal damage without loss of function^44^, commonly termed ‘subclinical AKI’^45^. Elevation of KIM-1 or NGAL without increases in serum creatinine was predictive of need for renal replacement therapy and in-hospital mortality in adult emergency admissions^46^ and for 3-year mortality in adults following cardiac surgery^47^. Our results suggest that repeated episodes of subclinical AKI may occur during aminoglycoside exposure leading to elevation of baseline KIM-1 suggestive of chronic tubular injury. As previously discussed, we are not able to make conclusions from these data about the predictive value of a rise in KIM-1 or NGAL for AKI, or to suggest any threshold value for the avoidance or withdrawal of aminoglycoside therapy. Assessment of the predictive value would require the measurement of KIM-1 and NGAL in large, prospective, observational cohort studies in children and adults receiving aminoglycoside therapy, using standardised AKI definitions^13^ and phenotypic criteria^48^, and powered for appropriate outcomes (AKI, renal replacement therapy, mortality).

In summary, we have demonstrated that in children and young adults with CF, significant changes occur in the urinary biomarkers KIM-1 and NGAL acutely during exposure to tobramycin. In addition, baseline KIM-1 concentration increases in line with cumulative exposure to aminoglycosides suggest chronic renal damage. KIM-1 in particular therefore holds potential as a biomarker of acute and chronic proximal tubular injury associated with exposure to aminoglycosides. The clinical utility of KIM-1 should be further evaluated in prospective studies. However, as a biomarker of subclinical AKI, it has immediate potential for use as a surrogate outcome marker in clinical trials looking at interventions to treat or prevent aminoglycoside-induced nephrotoxicity.

## Methods

### Patient recruitment and sample collection

The URBAN CF study (URinary Biomarkers of Aminoglycoside-induced Nephrotoxicity in children with Cystic Fibrosis) received ethical approval from the National Research Ethics Service Committee Northwest – Liverpool East, UK. It was registered on the UK Clinical Research Network portfolio (UKCRN 11815). The study was conducted in accordance with the Declaration of Helsinki. All methods were carried out in accordance with the relevant guidelines and regulations. Children and young adults with cystic fibrosis and their parents/guardians were recruited by a member of the research team between May 2012 and May 2013 through paediatric CF clinics at ten hospital sites in North-West England and Wales. Individuals up to 20 years of age with a confirmed diagnosis of CF (established by sweat test or genotype) were eligible. There were no exclusion criteria. Informed written consent was obtained from carers or guardians on behalf of the minors/children involved in our study. Participants above the age of 16 years were able to consent for themselves. All study data were collected in paper case report forms, and then entered into a secure electronic database.

Each subject was asked to provide a urine sample at the CF clinic on the day of recruitment, and then on each subsequent clinic visit for the duration of the study. Follow-up was for a minimum of 12 months and a maximum of 24 months (patients were recruited over a period of 12 months, and all participants were followed up for a further 12 months after the final patient was recruited). If subjects received one or more courses of treatment with intravenous (IV) tobramycin during this period, urine samples were collected regularly during the treatment course.

In the UK, CF patients can be offered IV treatment as either an inpatient or at home. In subjects receiving a course of tobramycin as an inpatient or at home with daily children’s community nurse visits, a baseline urine sample was collected from the patient on the day of, but prior to commencing, the course of treatment with tobramycin (day 0 sample). Further urine samples were collected each day, for the duration of the tobramycin treatment course. A further sample was collected 5–10 days after completion of the treatment course.

In subjects receiving a course of tobramycin at home without daily community nurse visits, a baseline urine sample was collected from the patient on the day of, but prior to commencing, the course of treatment with tobramycin (day 0 sample). Further samples were collected when the patient had their routine monitoring blood tests done. These usually occurred on day 1 (before the second dose of tobramycin) and on day 8 (before the 9^th^ dose of tobramycin). However, some patients had more frequent monitoring blood tests, or not on the days specified, and urine samples were collected on each day that monitoring blood tests were done. Another urine sample was collected on the final day of the course of tobramycin treatment (usually day 13, but courses varied in length). A further sample was collected 5–10 days after completion of the treatment course.

Urine samples were collected from each participant by an appropriate method dependent on their age. The normally preferred method was a clean catch urine sample into a sterile container. In younger children, samples were collected by placing cotton wool balls into the nappy. Samples were transferred to (if not already collected in) a sterile container and then transported to the local hospital laboratory. Here samples were centrifuged at 2000g for 4 min, and then the supernatant was aliquoted and stored at −80 °C within 4 hours of collection^49^.

No additional blood samples or investigations were performed as part of this study. Results of blood investigations done as part of routine clinical care were recorded for each patient. These included serum urea & creatinine, and serum tobramycin levels (all measured in local hospital laboratories).

### Determination of urinary biomarkers

Collected urine samples were thawed, mixed and centrifuged (3000 rpm, 5 min). Biomarker measurements were performed on the resulting supernatants. Urinary KIM-1 and NGAL were measured using validated electrochemiluminescent assays (Meso Scale Discovery (MSD), US)^16^. Urine samples were run in duplicate at a dilution of 1 in 10 in MSD Diluent 37, and repeated at a dilution of 1 in 100 if they remained too concentrated. Biomarker values were normalised to urinary creatinine which was determined spectrophotometically as previously described^50^. Normalised urinary biomarker values are presented as ng/mgCr. Laboratory analysis was blinded to participants’ clinical characteristics.

### Baseline biomarkers

The biomarker values measured in the first urine sample provided by each participant as part of the study were designated as the baseline values for this analysis. KIM-1 and NGAL were measured as described above, and corrected to urinary creatinine. Participants were not receiving intravenous aminoglycoside at the time of this baseline sample. The baseline serum creatinine value was the most recent serum creatinine concentration (µmol/l) recorded before the time of recruitment to the study.

Estimated GFR (eGFR) was calculated from baseline serum creatinine using the Schwartz formula^51^ following the methodology of Andrieux *et al*.^40^:

$$eGFR\,(Schwartz)=[k\ast height\,(cm)]/plasma\,creatinine\,(\mu mol/l)$$

where k = 40 under 2 years, k = 49 from 2 to 13 years, and above 13 years k = 62 for males and 49 for females.

### Sample size

Prior studies suggested a sample size of 40 children receiving aminoglycosides would be required^52^. A clinical feasibility survey at Alder Hey Children’s Hospital, Liverpool, UK, suggested that if 160 children with CF were recruited, 40 would receive at least one course of treatment with tobramycin during the study period.

### Statistical analysis

All statistical analyses were undertaken in R version 3.2.0^53^. To explore the association between age, gender, previous exposure to IV aminoglycoside and eGFR and baseline biomarker values, a multiple linear regression model was fitted with these variables as covariates and log-baseline biomarker value as outcome. Stepwise variable selection was applied using the R function ‘stepAIC’ to achieve a final regression model. For the analysis of serum creatinine as the outcome, eGFR was excluded as a covariate in the regression model (as serum creatinine is used in its calculation). A multiple linear regression model was also used to test for association between age, gender, previous exposure to IV aminoglycoside, baseline KIM-1 and baseline NGAL and eGFR, again with stepwise variable selection applied.

For all variables retained in each of the final models, the p-value, regression coefficient and 95% confidence interval, and r-squared value were extracted, the latter to provide an estimate of the proportion of variability in outcome explained by the variable.

The change in biomarker concentration (KIM-1, NGAL, and serum Creatinine) during exposure to tobramycin was described using a fold-change in the biomarker concentration by dividing the peak value on tobramycin by the pre-tobramycin (day 0) value, or, where a day 0 value was not available, the most recent prior value. AKI was defined as an increase in serum creatinine by 50% or more from baseline, as per the KDIGO criteria^13^. To explore the effect of aminoglycoside exposure on KIM-1 and NGAL levels, profile plots were prepared for the first exposure to tobramycin in this study for each individual, showing variability per individual across time. A mean line was also fitted using a locally weighted regression (lowess) to demonstrate overall trend.

### Data availability

The datasets generated and analysed during the current study are available from the corresponding author on reasonable request.
